# Mesoporous Li_3_VO_4_/C Submicron‐Ellipsoids Supported on Reduced Graphene Oxide as Practical Anode for High‐Power Lithium‐Ion Batteries

**DOI:** 10.1002/advs.201500284

**Published:** 2015-10-01

**Authors:** Qidong Li, Qiulong Wei, Jinzhi Sheng, Mengyu Yan, Liang Zhou, Wen Luo, Ruimin Sun, Liqiang Mai

**Affiliations:** ^1^ State Key Laboratory of Advanced Technology for Materials Synthesis and ProcessingWuhan University of TechnologyWuhan430070P.R. China

**Keywords:** anode, in situ XRD, Li_3_VO_4_, lithium‐ion battery, mesoporous

## Abstract

Despite the enormous efforts devoted to high‐performance lithium‐ion batteries (LIBs), the present state‐of‐the‐art LIBs cannot meet the ever‐increasing demands. With high theoretical capacity, fast ionic conductivity, and suitable charge/discharge plateaus, Li_3_VO_4_ shows great potential as the anode material for LIBs. However, it suffers from poor electronic conductivity. In this work, we present a novel composite material with mesoporous Li_3_VO_4_/C submicron‐ellipsoids supported on rGO (LVO/C/rGO). The synthesized LVO/C/rGO exhibits a high reversible capacity (410 mAh g^−1^ at 0.25 C), excellent rate capability (230 mAh g^−1^ at 125 C), and outstanding long‐cycle performance (82.5% capacity retention for 5000 cycles at 10 C). The impressive electrochemical performance reveals the great potential of the mesoporous LVO/C/rGO as a practical anode for high‐power LIBs.

## 1 Introduction

Nowadays, lithium‐ion batteries (LIBs) have monopolized the market of power supplies for portable electronics. Actually, the application of LIBs has been extended from portable electronics to electric vehicles, hybrid electric vehicles, and large‐scale energy storage systems.^1^ To power the large‐scale devices, LIBs with higher energy density and power density are urgently required. However, the most widely used graphite anode material in current commercial LIBs faces several obstacles: (1) the low power density because of its poor ionic conductivity; (2) the formation of solid‐electrolyte interphase (SEI) layer arising from the irreversible electrolyte decomposition during the initial cycle; (3) the safety issues caused by the lithium dendrites formation on graphite.^2^, ^3^, ^4^, ^5^ Therefore, it is an urgent demand to develop alternative anode materials for high‐power and safe LIBs.

**Figure 1:**
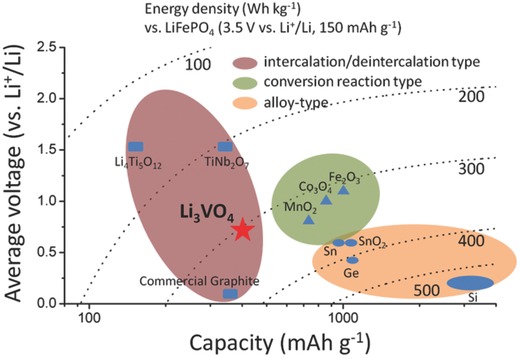
The average voltage versus capacity for the three types of anode materials, assuming LiFePO_4_ as the cathode material.

Up to now, three types of anode materials have been developed for LIBs: the alloy‐type (Si, Sn, SnO_2_, Ge, etc.),^6^, ^7^, ^8^ the conversion reaction type (Fe_2_O_3_, Co_3_O_4_, MnO_2_, etc.),^9^, ^10^, ^11^, ^12^ and the intercalation/deintercalation type (graphite,^13^ Li_4_Ti_5_O_12_,^4^ TiNb_2_O_7_,^14^ etc.). The distribution of their energy densities is illustrated in **Figure** **1**. The energy density is calculated by the following equation (Equation (1))

(1) $$E=\frac{\Delta V\times C}{1+m_{c}}$$

where *E* is the energy density, *∆V* is the operating voltage of the full cell, *C* is the capacity of the anode material, and *m*_c_ is the matching weight of the cathode material (LiFePO_4_ is assumed as the cathode material in this case).

The alloy‐type and conversion reaction type anode materials deliver high capacity. However, the huge volume changes during discharge/charge processes usually induce fast capacity fading. Besides, the low initial Coulombic efficiency (CE) of both types of anodes always arouses the wastage of Li^+^ from the expensive cathodes, hindering their practical applications. The intercalation/deintercalation type anode materials are regarded as the ideal choice for practical applications. The intrinsic advantages of intercalation/deintercalation anode materials, such as highly reversible Li^+^ insertion/extraction processes, usually result in good cycling stability. Li_4_Ti_5_O_12_, for example, has been extensively studied due to its excellent cycle life and rate capability.^15^, ^16^ Unfortunately, its limited theoretical capacity (175 mAh g^−1^) and relatively high operating voltage (≈1.5 V) restrict the output energy density of the assembled full cell. Recently, TiNb_2_O_7_ has been considered as another promising candidate with considerable capacity (theoretical capacity of 387 mAh g^−1^) and safe operation.^14^, ^17^ However, it still suffers from high operating voltage (above 1.0 V). As a result, the energy density of TiNb_2_O_7_ is quite limited, as plotted in Figure 1.

Encouragingly, Li_3_VO_4_ (denoted as LVO), a solid ionic conductor, delivers a high theoretical capacity of 394 mAh g^−1^, corresponding to the insertion of two Li^+^ per formula during lithiation. The working voltage of LVO (0.5–0.8 V vs Li^+^/Li) is lower than those of Li_4_Ti_5_O_12_ and TiNb_2_O_7_, which may bring higher energy density in Li‐ion full cell. Meanwhile, it is higher than that of the graphite, which may avoid the safety issue of short‐circuiting associated with the formation of Li dendrites. Moreover, the high ionic conductivity of LVO (≈10^−6^ S cm^−1^)^18^ may ensure the rapid diffusion of Li^+^ in the crystal structure, leading to good rate performance. These advantages make the LVO one of the most promising candidates among currently studied anode materials.^19^, ^20^, ^21^, ^22^, ^23^, ^24^, ^25^, ^26^, ^27^, ^28^ However, some barriers still need to be conquered before its wide application. Compared to the ionic conductivity, the electronic conductivity of LVO is poor, which would arouse large over potential between the discharge/charge processes and deteriorate the rate capacity. Many approaches, such as carbon coating, modifying with rGO, and compositing with carbon nanotubes, have been implemented to improve the electronic conductivity as well as the rate capability of LVO.^19^, ^20^, ^21^, ^23^, ^24^, ^27^, ^28^ Although these methods show decent boost in performance, further improvement in electrochemical performance is desirable. For example, the tap density and volumetric density of hollow structured Li_3_VO_4_ prepared by hydrothermal method should be improved; the initial CE of Li_3_VO_4_/graphene and Li_3_VO_4_/C nanocomposites should be enhanced.

According to the formula *t* ~ *L* ^2^/*D*, the diffusion time (*t*) for Li^+^ is related to the diffusion length (*L*) and the diffusion coefficient (*D*). Reducing the particle size down to nanoscale is an effective strategy to improve the rate performance.^29^ However, the smaller the particle size, the more serious the agglomeration. In this regard, constructing porous structures, especially mesoporous structures, is preferred. The mesoporous structure is able to enlarge the electrode–electrolyte contact area, provide more active sites for Li^+^ storage, and provide mesoporous channels for rapid Li^+^ diffusion.^14^, ^17^, ^30^, ^31^ In addition, the mesoporous structure offers free space to accommodate the volume change caused by repeated Li^+^ intercalation/deintercalation, enhancing the structural stability. However, the synthesis of mesoporous LVO has not been reported due to the quick crystal growth of LVO under hydrothermal and solid state reaction conditions.^4^, ^19^, ^20^, ^22^

Herein, for the first time, we report a novel LVO/C/rGO nanocomposite with mesoporous Li_3_VO_4_/C submicron‐ellipsoids supported on rGO. The LVO/C/rGO composite shows a considerably high specific capacity with outstanding rate performance and cycling stability. The excellent electrochemical performance makes the mesoporous LVO/C/rGO composite a promising practical anode for high‐power LIBs.

## 2 Results and Discussion

**Figure 2:**
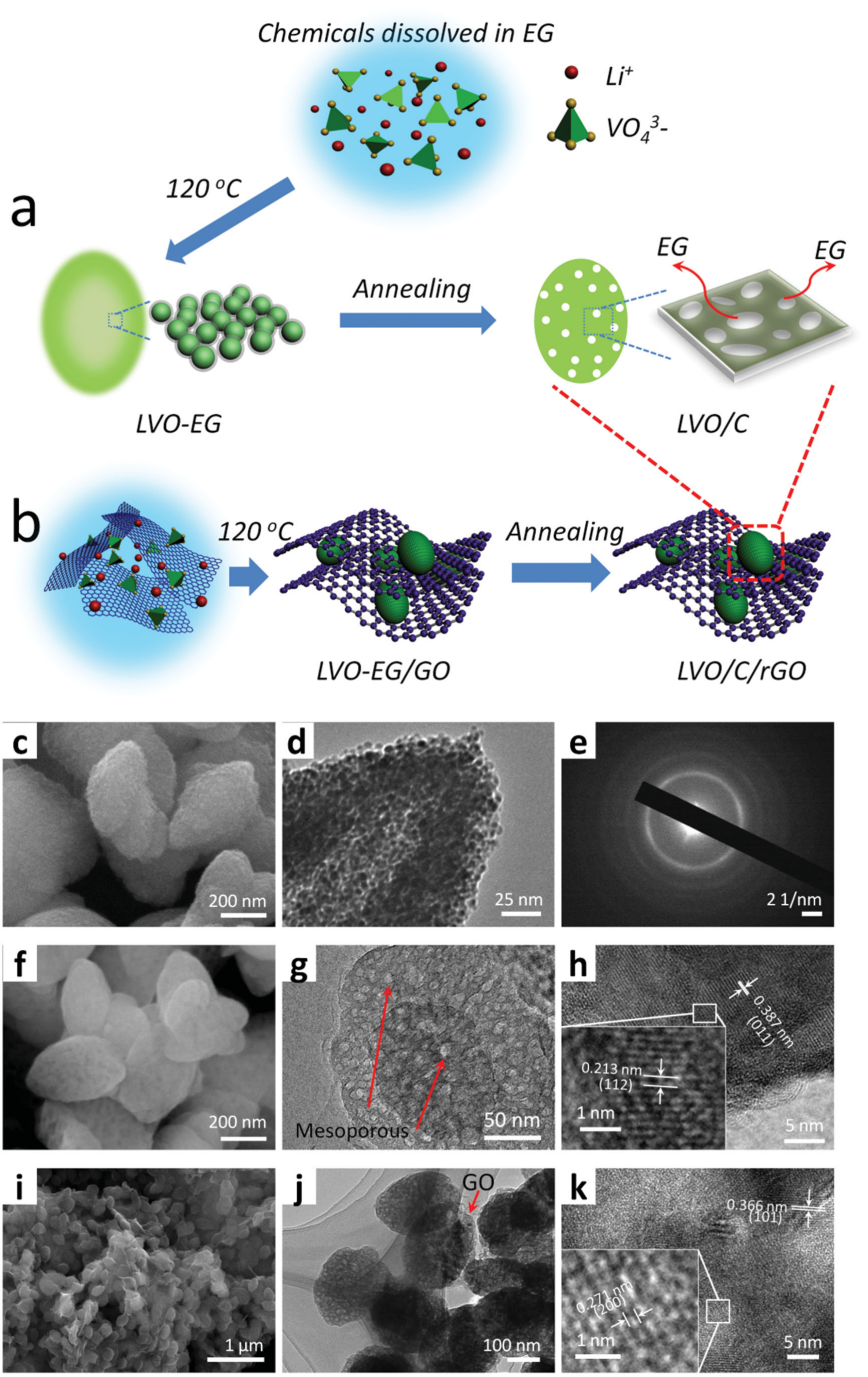
a) The proposed formation mechanism of the mesoporous LVO/C. b) Schematic illustration for the synthesis procedure of the LVO/C/rGO. c) SEM image, d) TEM image, and e) SAED pattern of the LVO‐EG. f) SEM, g) TEM, and h) HRTEM images of LVO/C. i) SEM, j) TEM, and k) HRTEM images of LVO/C/rGO.

The mesoporous LVO/C nanocomposite is synthesized through a facile and scalable solution method followed by carbonization (**Figure** **2**a). The reaction between LiOH·H_2_O and NH_4_VO_3_ in solution leads to the formation of LVO nanoclusters with ethylene glycol (EG) adsorbed on the surface. To reduce the surface energy, the LVO‐EG nanoclusters further self‐assemble into submicron‐ellipsoids.^32^ The subsequent annealing of LVO‐EG submicron‐ellipsoids in Ar causes the volatilization, decomposition, and carbonization of EG, leaving a significant amount of mesopores in the obtained LVO/C. To enhance the electronic conductivity of LVO, graphene oxide (GO) nanosheets are introduced during the synthesis (Figure 2b). Due to the existence of functional groups, the GO nanosheets wrapped uniformly on the LVO‐EG submicron‐ellipsoids, forming LVO‐EG/GO composite. During annealing, the GO is reduced to rGO, the EG is decomposed into carbon, and the LVO/C/rGO is obtained.

Figure 2c–k and Figure S1 (Supporting Information) show the morphology and structure of the samples at different states. The particle size of LVO‐EG submicron‐ellipsoids is about 300 nm in length and 200 nm in width (Figure 2c). The transmission electron microscopy (TEM) image (Figure 2d) shows that the submicron‐ellipsoids are composed of small nanocrystallines with a size of 2–5 nm. The formation of small nanocrystallines is due to the restricted crystal growth of LVO by the adsorbed EG. The selected area electron diffraction (SAED) pattern of an individual LVO‐EG submicron‐ellipsoid (Figure 2e) shows clear diffraction rings, indicating the nanocrystalline feature of the as‐synthesized sample. After annealing, the color of the sample changes from light yellow (LVO‐EG) to gray‐black (LVO/C) (Figure S2a, Supporting Information). The obtained LVO/C submicron‐ellipsoids show noticeable mesoporosity (Figure 2g). The thermogravimetric analysis (TGA) result shows a 10.2% weight lost during annealing in Ar (Figure S2b, Supporting Information), indicating that the in situ carbonization of EG is the main reason for the formation of mesopores.^9^, ^12^ The introduction of GO does not affect the morphology and the chemical composition of LVO‐EG submicron‐ellipsoids (Figure S1, Supporting Information). As shown in Figure 2i, the LVO/C submicron‐ellipsoids are uniformly supported on the rGO. Meanwhile, the LVO/C/rGO exhibits a mesoporous structure similar to LVO/C (Figure 2j,k).

**Figure 3:**
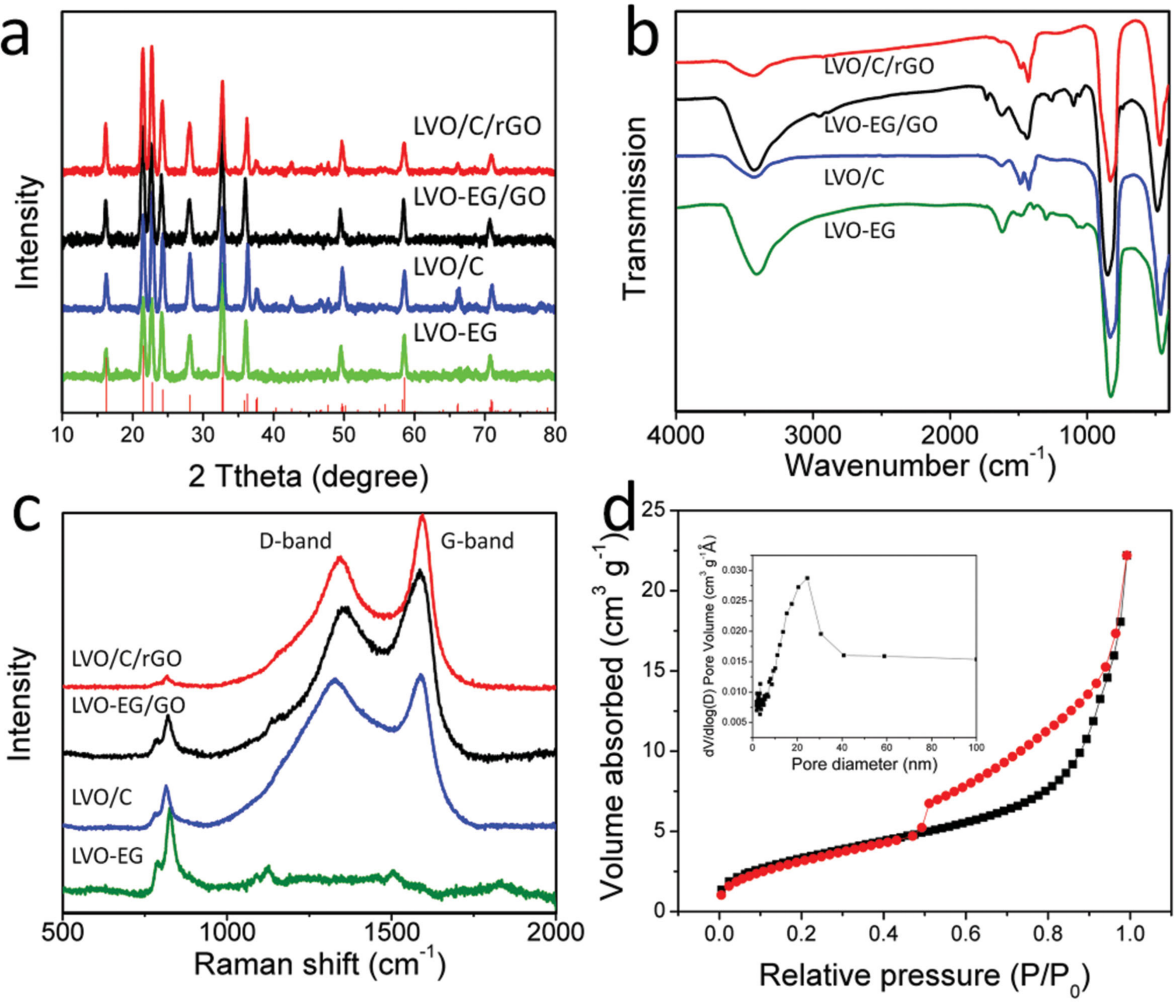
a) The XRD patterns, b) FT‐IR spectra, and c) Raman spectra of LVO‐EG, LVO/C, LVO‐EG/GO, and LVO/C/rGO. d) Nitrogen adsorption–desorption isotherm and pore size distribution of LVO/C/rGO.

X‐ray diffraction (XRD) patterns of the samples are illustrated in **Figure** **3**a. The XRD pattern of LVO‐EG can be indexed to the orthorhombic Li_3_VO_4_ phase (JCPDS card No. 38‐1247). After the annealing process, several diffraction peaks shift to high angle, indicating the shrinkage of the crystal (Figure 3a and Figure S3, Supporting Information). Among the (100), (002), and (040) diffraction peaks, the (002) peak shows the most obvious right‐shift, suggesting the largest contraction along *c*‐axis (Figure S3, Supporting Information). A similar trend can be observed in the LVO‐EG/GO and LVO/C/rGO (Figure 3a). Rietveld refinement of the XRD spectra is used to analyze the exact change in lattice parameters. The results show that, during annealing, the *a*, *b*, *c* change from 6.309, 5.452, and 4.972 to 6.310, 5.446, and 4.940 Å, respectively (Table S1, Supporting Information). Overall, the cell volume reduces by 0.74% after annealing.

Fourier transform infrared (FT‐IR) spectra of the samples are shown in Figure 3b and their assignment is summarized in Table S2 (Supporting Information). The two strong characteristic peaks of LVO, which are located at 840 and 470 cm^−1^, can be observed in all samples.^33^ For LVO‐EG, the bands detected at 1638, 1404, and 1314 cm^−1^ are assigned to the —OH bending, —CH_2_— deformation, and —CH_2_— in‐plane bending of EG, respectively.^34^ When GO is introduced into LVO‐EG, some new peaks appear at 2949, 1721, 1246, 1088, and 1046 cm^−1^, corresponding to the —CH_2_CH_2_— stretching, carboxyl group vibration, ether C—O vibration, phenolic C—O vibration, and alcoholic C—O vibration.^34^, ^35^ After annealing in Ar, the FT‐IR spectra confirm the volatilization and decomposition of EG and the removal of functional groups of GO.

Raman spectrum of the LVO/C (Figure 3c) shows two characteristic bands for carbonaceous materials: the D‐band (disorder‐induced phonon mode) and the G‐band (graphite band). By a Gaussian numerical simulation, the two broad peaks (D‐band and G‐band) can be decomposed into four peaks.^36^ The peaks around 1350 cm^−1^ and 1590 cm^−1^ are correlated with sp^2^‐type carbon, while the other two peaks around 1180 cm^−1^ and 1510 cm^−1^ are relevant to sp^3^‐type carbon. A low integrated area ratio of sp^3^ to sp^2^ (*A*_sp_3/*A*_sp_2) indicates that a large fraction of carbon exists as the sp^2^ type. The fitted *A*_sp_3/*A*_sp_2 values of the LVO/C, LVO‐EG/GO and LVO/C/rGO are 0.518, 0.728, and 0.389, respectively (Figure S4, Supporting Information), suggesting the reduction of GO during annealing. The peak area ratios of D band to G band (the *I*_D_/*I*_G_ ratio) are correlated to the size of the ordered domains (*d*_dom_) of graphitic samples.^34^ The values of *I*_D_/*I*_G_ and *d*_dom_ are summarized in Table S3 (Supporting Information). The *d*_dom_ of LVO/C/rGO is determined to be 17.9 nm.^34^ The elemental analysis results confirm that the amount of carbon in the LVO/C and LVO/C/rGO is 2.62% and 6.04%, respectively. The content of rGO in LVO/C/rGO is determined to be 3.42% in weight, which is quite small compared to other carbon composite materials.^37^, ^38^

Nitrogen sorption is carried out to analyze the surface area and porosity of the samples (Figure 3d and Figure S5, Supporting Information). As expected, the solid LVO particles exhibit very low Brunauer–Emmett–Teller (BET) surface area and total pore volume (Table S4, Supporting Information). The LVO/C/rGO and LVO/C display a type II isotherm with an H_3_ hysteresis loop, which is typical for mesoporous materials with irregular pore systems.^39^ Relatively broad mesopores centered at around 25 nm can be observed in the pore size distribution curves of LVO/C/rGO and LVO/C (Figure 3d and Figure S5b, Supporting Information), which agrees well with the TEM observations. As a result, the LVO/C/rGO and LVO/C show much higher BET surface area and pore volume than the solid LVO particles (Table S4, Supporting Information).

**Figure 4:**
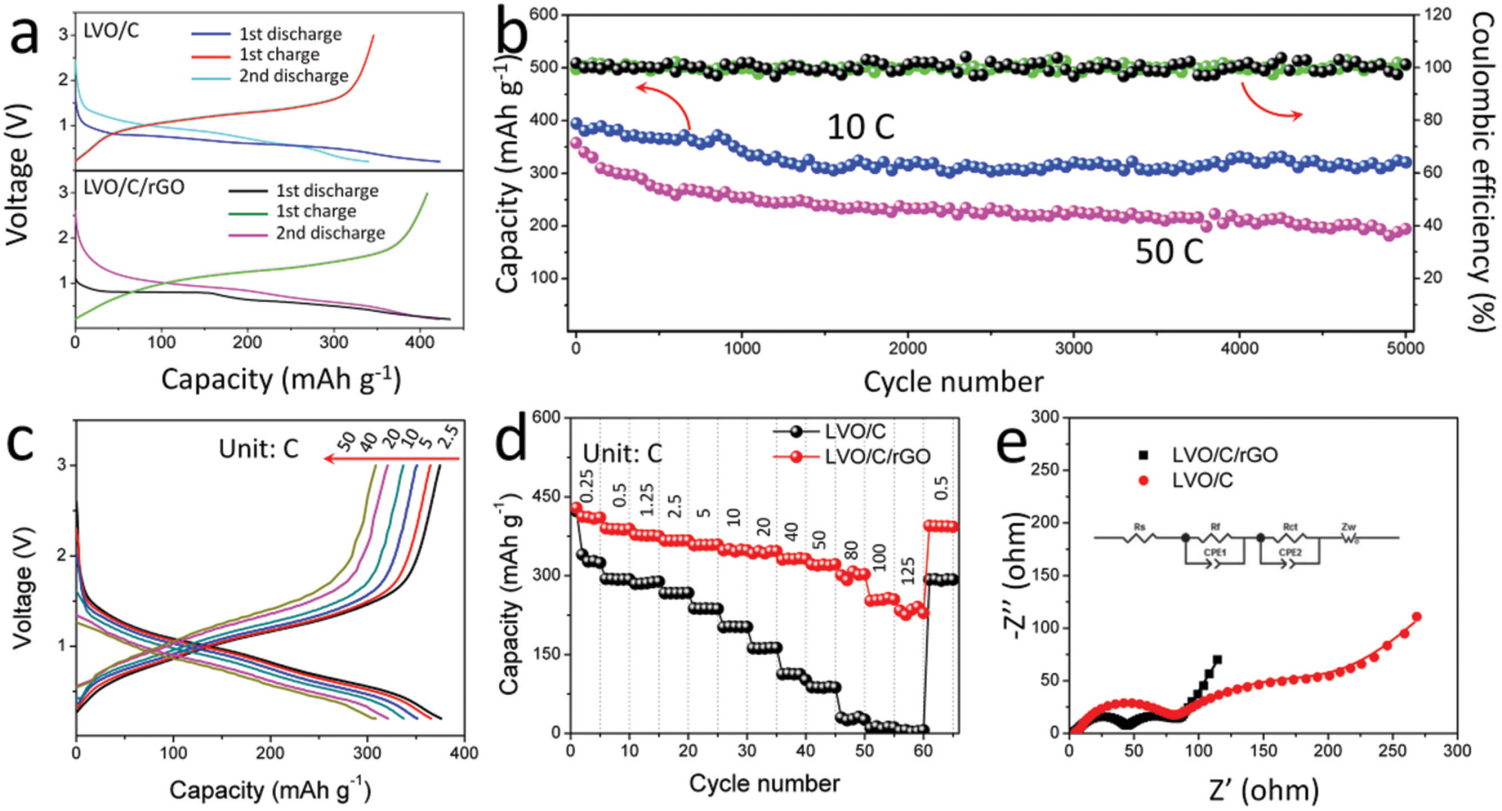
a) The first and second discharge/charge curves for the LVO/C and LVO/C/rGO at 0.1 A g^−1^. b) Cycling performance of LVO/C/rGO at 4 and 20 A g^−1^. c) Discharge/charge curves of LVO/C/rGO at different current densities. d) Rate performances of the LVO/C and LVO/C/rGO. e) EIS spectra of LVO/C and LVO/C/rGO after 25 cycles.

The electrochemical performances are evaluated by assembling coin cells (2016‐type) with Li foil as the counter electrode. All cells measurements are carried between 0.2 and 3.0 V versus Li^+^/Li at room temperature. The specific capacities are calculated based on the total mass of the composites (including the rGO and amorphous carbon). **Figure** **4**a shows the first two discharge/charge voltage curves of the LVO/C and LVO/C/rGO at 0.25 C (1 C = 400 mA g^−1^). The initial discharge capacities of LVO/C and LVO/C/rGO are 423 and 435 mAh g^−1^, respectively. According to the voltage profiles, about 80% of the specific capacity is obtained between 0.5 and 1.5 V. The initial CE for LVO/C is 81.9%. With the introduction of rGO, the initial CE increases to 94.0%, indicating the high reversibility of the electrochemical reaction during charge/discharge. The initial CE of LVO/C/rGO is significantly higher than those of alloy type and conversion reaction type anode materials, which is usually lower than 75%. The second discharge capacity of LVO/C/rGO is 412 mAh g^−1^, slightly higher than the theoretical capacity (394 mAh g^−1^). The excess discharge capacity may be attributed to the capacity contribution from rGO and the capacitive behavior of nanosized electrode materials.^21^, ^40^

Figure 4b shows the cycling performance and corresponding CE of LVO/C/rGO. At a rate of 10 C (4 A g^−1^), the LVO/C/rGO delivers an initial capacity of 394 mAh g^−1^, maintaining 325 mAh g^−1^ after 5000 cycles (82.5% of its initial capacity). At a higher rate of 50 C (20 A g^−1^), an initial discharge capacity of 357 mA g^−1^ can be achieved. After 5000 cycles, a capacity of 200 mA h g^−1^ can be retained, corresponding to a capacity fading of 0.011% per cycle. The CE is around 100% during the whole testing processes. The high CE along with the outstanding long‐cycle performance unambiguously demonstrates the great potential of LVO/C/rGO as practical anode for high‐power LIBs. Without the rGO, the LVO/C shows a capacity below 210 mAh g^−1^ at 10 C (Figure S6a, Supporting Information).

For large‐scale devices, high power density is indispensable. A very impressive rate performance is obtained for the LVO/C/rGO (Figure 4c,d). For the rate performance, the discharge/charge currents are varied from 0.25–125 C (0.1 to 50 A g^−1^). The LVO/C/rGO delivers a capacity of 410, 377, 358, 345, 320, and 230 mAh g^−1^ at a rate of 0.25, 1.25, 5, 20, 50, and 125 C, respectively. At a high current density of 125 C (50 A g^−1^), the full charge or discharge process can be completed within 17 s. Remarkably, no significant change in the voltage profiles is observed when the current density is increased from 2.5 to 50 C (Figure 4c). In contrast, the LVO/C shows an enlarged over potential with the increase of the current density (Figure S6b, Supporting Information). For comparison, the rate performance of solid LVO/rGO has been provided in Figure S7 (Supporting Information). As expected, the solid LVO/rGO exhibit significantly inferior rate performance than LVO/C/rGO, demonstrating the positive effect of mesoporous structure on rate performance. The lithium storage performances of recently published LVO‐based anode materials are summarized in Table S5 (Supporting Information). From Table S5 (Supporting Information), it is safe to draw the conclusion that our material (LVO/C/rGO) shows better cycling stability and rate capability than other LVO‐based anode materials reported in literatures.^19^, ^20^, ^21^, ^22^, ^23^, ^24^, ^25^, ^26^, ^27^, ^28^ A statistical power density distribution of the state‐of‐the‐art intercalation/deintercalation anodes is illustrated in Figure S8 (Supporting Information), where LiFePO_4_ is assumed to be the cathode material. The LVO/C/rGO exhibits a very impressive power density, which is about two times to those of Li_4_Ti_5_O_12_ and TiNb_2_O_7_. For practical applications, the amount of carbon needs to be reduced. Electrodes with an active material:acetylene black:binder ratio of 92:3:5 have been prepared and the LVO/C/rGO delivers a capacity of 395, 318, and 280 mAh g^−1^ at 0.25, 10, and 40 C, respectively (Figure S9, Supporting Information). This impressive result makes the LVO/C/rGO a very promising anode material for practical applications.

The electrochemical impedance spectrum (EIS) measured from 0.01 Hz–100 kHz is presented in Figure 4f. After simulation, the charge transfer resistance (*R*_ct_) values for the LVO/C and LVO/C/rGO after 25 discharge–charge cycles are calculated to be 117.6 and 34.3 Ω, respectively. The smaller charge transfer resistance of the LVO/C/rGO makes it more favorable for high‐rate performance.

**Figure 5:**
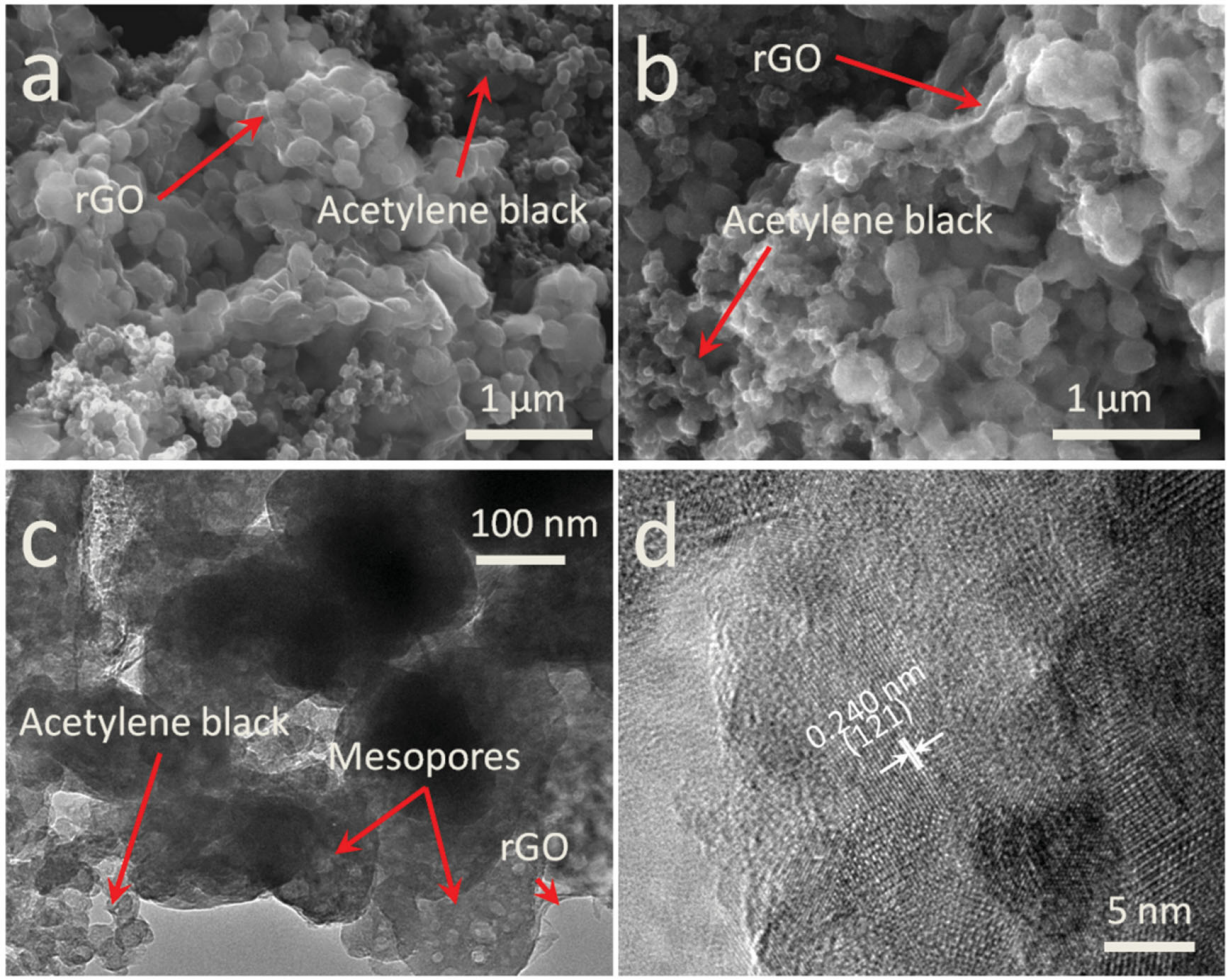
a) SEM image of LVO/C/rGO before cycling. b) SEM, c) TEM, and d) HRTEM images of LVO/C/rGO after 1000 discharge–charge cycles.

To study the structural stability, the SEM and TEM images of LVO/C/rGO before and after 1000 cycles are collected. No obvious morphology difference can be observed in SEM; for both samples, the LVO/C submicron‐ellipsoids are well wrapped with the rGO (**Figure** **5**a,b). The mesoporous structure is well maintained after long‐term cycles (Figure 5c), indicating the excellent structural stability of LVO/C/rGO. The high‐resolution TEM (HRTEM) image (Figure 5d) shows clear lattice fringes from LVO, which further demonstrates the superior structural stability.

**Figure 6:**
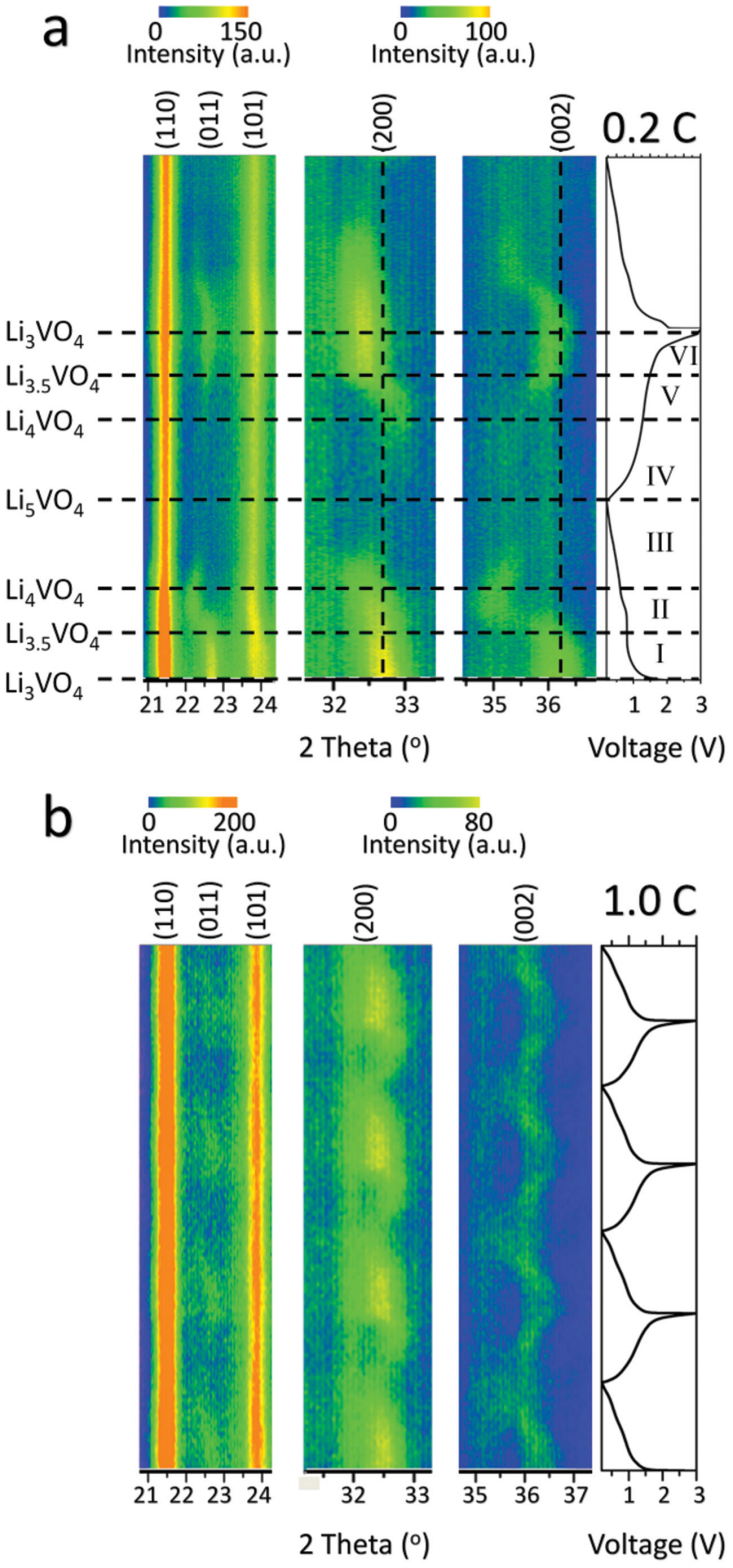
In situ XRD patterns collected during galvanostatic discharge/charge of the LVO/C/rGO half‐cell. a) The image plot of diffraction patterns for (110), (011), (101), (200), and (002) reflections during the first two discharge/charge cycles at 80 mA g^−1^ (0.2 C) and b) the following discharge/charge cycles at 400 mA g^−1^ (1.0 C).

The lithium storage mechanism of the LVO/C/rGO upon electrochemical discharge/charge processes is further investigated by in situ XRD. In this testing, we used an advanced area detector, which is able to collect the XRD reflections with a wide 2*θ* range (about 25°) simultaneously. As illustrated in **Figure** **6**a, clear contour plot of peak intensities versus operating states displays the phase transition process at 80 mA g^−1^ (0.2 C). Selected acquisition windows are chosen to in situ monitor the (110), (011), (101), (200), and (002) diffractions during the discharge/charge processes.^29^, ^41^ These diffractions are very sensitive to the lithium storage process. All the diffractions at the open circuit voltage state can be assigned to the Li_3_VO_4_ phase. With the intercalation of ≈0.5 Li^+^ per formula (step I), the (011), (200), and (002) reflections slightly shift to lower 2*θ* angle, which is a typical single‐phase reaction.^22^, ^42^, ^43^ In the subsequent discharge process (step II, from Li_3.5_VO_4_ to Li_4_VO_4_), the (011) and (002) reflections disappear gradually along with the appearance of two new diffractions at 22.2° (denoted as (011)*) and 35.1° (denoted as (002)*), indicating the formation of a new phase. Meanwhile, the (101) and (200) diffractions shift to low angle and decrease in intensity. The large left shift of the (200) reflection indicates the increase of the *d*‐spacing along the *a‐*axis, which may facilitate the subsequent ion diffusion. With one more Li^+^ intercalation (step III, from Li_4_VO_4_ to Li_5_VO_4_), the (011)*, (200), and (002)* reflections gradually disappear. In the following charge process (steps IV–VI), these three reflections cannot be recovered until 1.5 Li^+^ are extracted from Li_5_VO_4_ (step V). At the end of charge process, all the peaks appearing in Li_3_VO_4_ can be recovered (step VI), demonstrating the high reversibility of the electrochemical reaction. However, the *d*_(200)_ and *d*_(002)_ have been expanded after one discharge–charge cycle when compared to the original Li_3_VO_4_. The expansion in *d*_(200)_ and *d*_(002)_ is beneficial for further cycling and might be the reason for the difference between the first and second discharge curves. In the whole processes (from steps I to VI), the (110) reflection shows little difference, indicating the high stability of LVO framework during Li^+^ insertion/extraction.^42^

The structural change of Li_3_VO_4_ at a relatively high charge–discharge current (400 mA g^−1^, 1C) is also monitored by in situ XRD (Figure 6b). The (110) and (101) reflections exhibit little change, suggesting the stable framework of LVO at high rate. Different from the results obtained at 0.2 C, the positions of (200) and (002) reflections shift continually, indicating a nonequilibrium solid solution phase (Li_3*+x*_VO_4_, 0 < *x* < 2) reaction mechanism. The nonequilibrium solid solution phase reaction is also highly reversible during cycling, which underpins the high‐rate capability.^29^

The superior electrochemical performances of LVO/C/rGO can be attributed to the following reasons. (1) The rGO largely enhances the electronic conductivity of LVO, which reduces the polarization during charge and discharge processes, especially at high rate. (2) The intrinsic ionic conductivity of LVO provides rapid Li^+^ diffusivity. Meanwhile, the mesoporous structure provides short Li^+^ diffusion pathway. Both factors may enhance the electrochemical reaction kinetics, leading to excellent high rate performance. (3) The robust framework makes the LVO an excellent host material for Li^+^ insertion/extraction, ensuring outstanding cycling performance. The enhanced electron/Li^+^ diffusion along with the stable LVO framework endows the LVO/C/rGO with outstanding high‐rate long‐cycle performance.

## 3 Conclusions

The mesoporous LVO/C/rGO nanocomposite has been successfully synthesized by a facile and scalable solution method followed by annealling process. As the LIBs anode, the mesoporous LVO/C/rGO composite displays a considerably high reversible capacity, excellent rate capability, and outstanding long‐cycle performance. The remarkable electrochemical performance is attributed to the intrinsic rapid ionic conductivity of LVO, the short Li^+^ diffusion pathway originated from the mesoporous structure, the enhanced electronic conductivity brought by the highly conductive rGO, and the robust framework. The impressive electrochemical performance unambiguously demonstrates that the mesoporous LVO/C/rGO composite is a very promising anode material for high‐power LIBs.

## 4 Experimental Section

*The Synthesis of Mesoporous LVO/C/rGO Nanocomposite*: GO was first dispersed in EG by ultrasonication. LiOH·H_2_O (70 mmol) and NH_4_VO_3_ (2 mmol) were then added into the GO dispersion by magnetic stirring and ultrasonication. The solution was kept in an oil bath at 120 °C under stirring for 30 min. The precipitates collected by centrifugation were washed with ethanol for several times and dried in an oven at 70 °C. The dry powder (LVO‐EG/GO) was annealed at 600 °C for 3 h in Ar (heating rate: 10 °C min^−1^) to obtain the mesoporous LVO/C/rGO. The LVO/C was synthesized using the same process except that no GO was introduced. The solid LVO and LVO/GO particle were synthesized by replacing EG with DI water.

*Material Characterization*: Field emission scanning electron microscopy images were collected with a JEOL‐7100F microscopy. TEM and HRTEM images were recorded by using a JEM‐2100F STEM/EDS microscope. Raman spectra were obtained using a Renishaw INVIA micro‐Raman spectroscopy system. FT‐IR spectra were obtained using a Nexus system. BET surface areas were measured using Tristar II 3020 instrument by nitrogen adsorption of at 77 K. XRD patterns of the samples were obtained with a D8 Advance X‐ray diffractometer, using Cu‐Kα radiation (*λ* = 1.5418 Å). For in situ XRD testing, an electrochemical cell module with a beryllium window was used, while the slurry was directly cast on the beryllium window. TGA was performed using a Netzsch STA 449C simultaneous analyzer. The element analyses were performed by an Elementar Vario EL cube elemental analyzer.

*Measurement of Electrochemical Performance*: The electrochemical properties were characterized in 2016 coin cells with lithium foils as the anode. The working electrodes were prepared by mixing the active materials, acetylene black, and carboxyl methyl cellulose at a weight ratio of 70:25:5. The slurry was cast onto Cu foil and dried in a vacuum oven at 150 °C for 2 h. The mass loading of active materials was 1–2 mg cm^−2^. The electrolyte is composed of 1 m LiPF_6_ dissolved in ethylene carbonate/dimethyl carbonate with a volume ratio of 1:1. Galvanostatic discharge/charge cycling behaviors were investigated with a multichannel battery testing system (LAND CT2001A). Electrochemical impedance spectroscopies (EIS) were tested with an Autolab Potentiostat Galvanostat. All the measurements were carried out at room temperature.

## Supporting information

As a service to our authors and readers, this journal provides supporting information supplied by the authors. Such materials are peer reviewed and may be re‐organized for online delivery, but are not copy‐edited or typeset. Technical support issues arising from supporting information (other than missing files) should be addressed to the authors.

Supplementary
