# Supplementary material for: Mesoporous Li3VO4/C Submicron‐Ellipsoids Supported on Reduced Graphene Oxide as Practical Anode for High‐Power Lithium‐Ion Batteries
Source: Adv Sci (Weinh). 2015 Oct 1;2(12):1500284. doi: 10.1002/advs.201500284 (PMC5054844; doi:10.1002/advs.201500284)
Supplement: Supplementary file 1 — Supplementary [file ADVS-2-0j-s001.pdf]

Copyright WILEY-VCH Verlag GmbH & Co. KGaA, 69469 Weinheim, Germany, 2015.

Supporting Information

for *Adv. Sci.*, DOI: 10.1002/advs.201500284

Mesoporous Li_3VO_4/C Submicron-Ellipsoids Supported on Reduced Graphene Oxide as Practical Anode for High-Power Lithium-Ion Batteries

Qidong Li, Qiulong Wei, Jinzhi Sheng, Mengyu Yan, Liang Zhou, Wen Luo, Ruimin Sun, and Liqiang Mai**

Copyright WILEY-VCH Verlag GmbH & Co. KGaA, 69469 Weinheim, Germany, 2015.

Supporting Information

Mesoporous Li_3VO_4/C Submicron-ellipsoids Supported on Reduced Graphene Oxide as Practical Anode for High-Power Lithium-Ion Batteries

Qidong Li, Qiulong Wei, Jinzhi Sheng, Mengyu Yan, Liang Zhou, Wen Luo, Ruimin Sun, and Liqiang Mai**

Q. D. Li, Q. L. Wei, J. Z. Sheng, M. Y. Yan, Prof. L. Zhou, W. Luo, R. M. Sun, Prof. L. Q. Mai
State Key Laboratory of Advanced Technology for Materials Synthesis and Processing, Wuhan University of Technology, Wuhan 430070, China.
E-mail: mlq518@whut.edu.cn; liangzhou@whut.edu.cn.

Keywords: lithium-ion battery; anode; mesoporous; high-power; Li_3VO_4

The SI file includes detailed Experimental Methods, 5 Tables and 9 Figures.

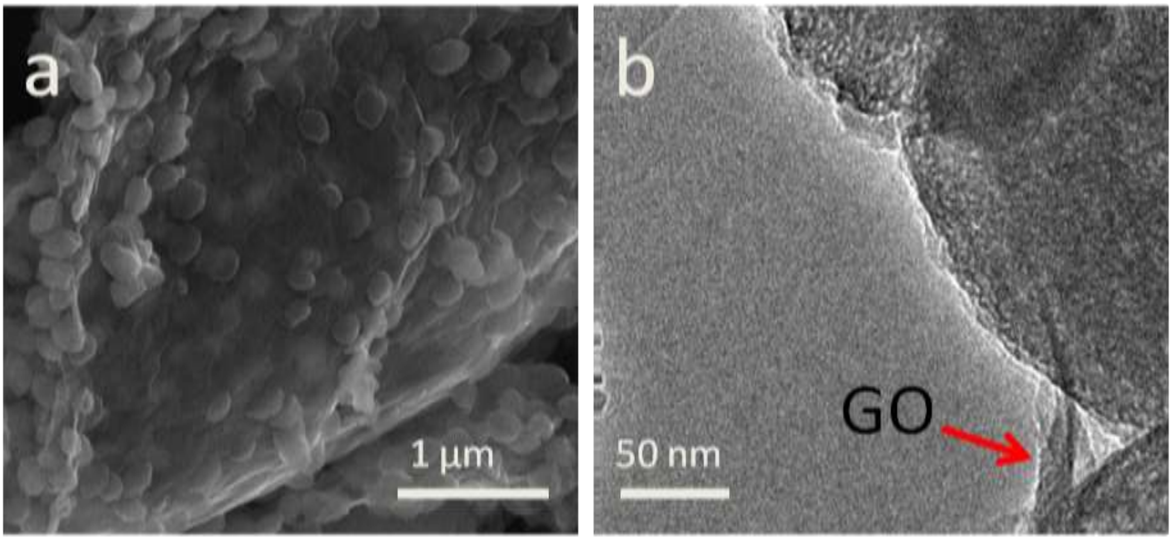

Figure S1. (a) SEM and (b) TEM images of LVO-EG/GO.

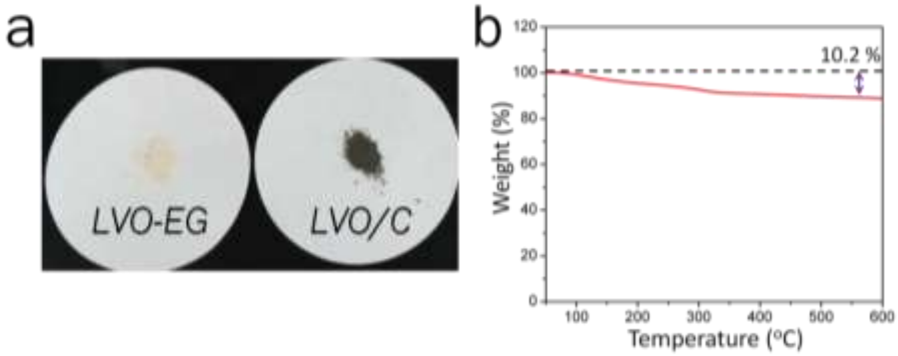

Figure S2. (a) Optical images of LVO-EG and LVO/C. (b) Thermogravimetric analysis (TGA) of LVO-EG in Ar atmosphere.

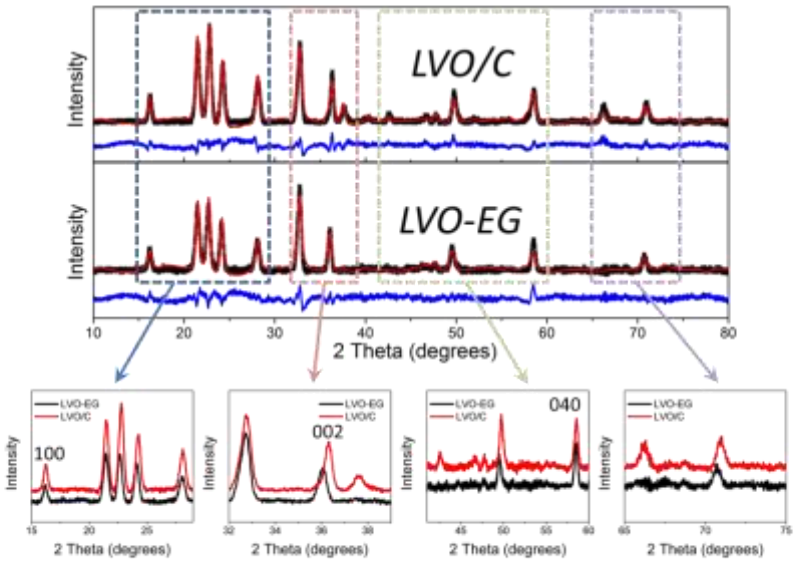

Figure S3. XRD patterns and the refinement XRD patterns of LVO/C and LVO- EG.

Table S1 Cell parameters of LVO-EG and LVO/C.

	a (Å)	*b* (Å)	*c* (Å)	cell volume ($Å^3$)
LVO-EG	6.309	5.452	4.972	171.020
LVO/C	6.310	5.446	4.940	169.759

Table S2 FT-IR band assignments of the four samples

LVO-EG	LVO/C	LVO-EG/GO	LVO/C/rGO	Band assignment	Reference
3428	3430	3420	3435	*v*(OH) water	34
		2949		*v*$(CH)_{as}$	34
		1721		*v*(COO)	35
1638		1622		*δ*(OH)	34
1496	1488	1478	1476	Amorphous carbon	34
	1425	1428	1426	Amorphous carbon	35
1404				(CH2) deformation	34
1314				*δ*(CH2) in-plane	34
		1246		*v*(C-O) ether	34
		1088		*v*(CO) phenolic	34
1047		1046		*v*(C-O) alcoholic	34
842	831	841	828	*v*(V-O-V)	33
472	464	476	465	*v*(V-O-V)	33

v: stretching; *δ*: bending.

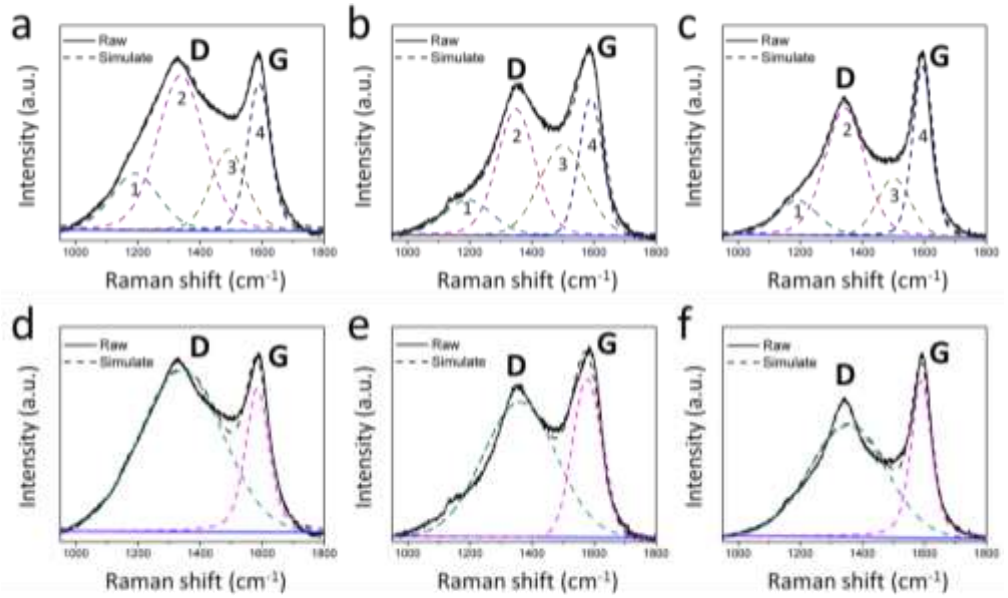

Figure S4. The Raman spectrum of LVO/C (a,d), LVO-EG/GO (b,e) and LVO/C/rGO (c,f).

Table S3 The I_D/I_G and d_{dom} values of LVO/C, LVO-EG/GO, and LVO/C/rGO.

	LVO/C	LVO-EG/GO	LVO/C/rGO
I_D/I_G	3.30	2.16	2.15
d_{dom} (nm)	11.676	17.839	17.922

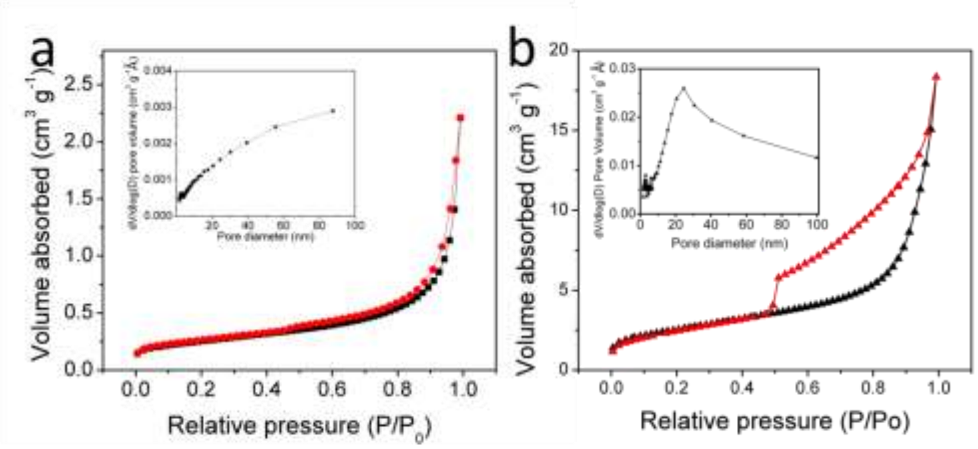

Figure S5. Nitrogen adsorption-desorption isotherms and pore size distribution curves of solid LVO particle (a) and LVO/C (b).

Table S4 The BET surface area and pore volume of the solid LVO, LVO/C, and LVO/C/rGO.

	solid LVO	LVO/C	LVO/C/rGO
BET surface area (m^2 g^{-1})	0.89	9.0	12.5
Pore volume (cm^3 g^{-1})	0.003	0.028	0.037

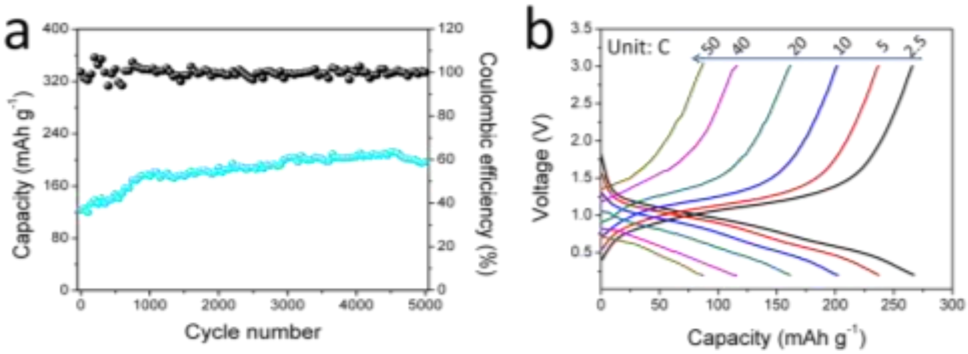

Figure S6. (a) Cycling performance of LVO/C anode at 10 C. (b) Discharge/charge curves of LVO/C anode at different current densities. (c) Rate performance of solid LVO/rGO electrode.

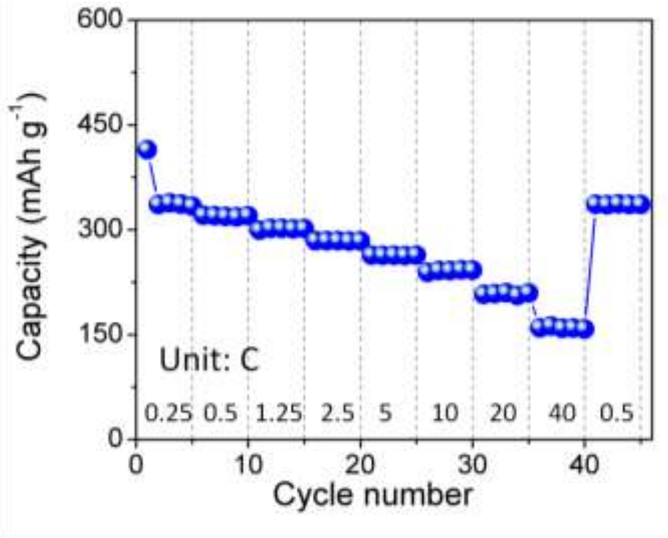

Figure S7. Rate performance of solid LVO/rGO electrode.

Table S5 The comparison of the electrochemical performances with precious works.

References	Anode	Cycling performance	Rate capability
Nanoscale, 2014, 6 11072	LVO/CNT	250 mAh g^{-1} after 2000 cycles at 5 C	240 mAh g^{-1} at 40 C
Chem. Commun. 2015, 51, 229	LVO@GNS	163 mAh g^{-1} after 5000 cycles at 5 C	133 mAh g^{-1} at 50 C
Nano Lett. 2013, 13, 4715	LVO/G	215 mAh g^{-1} after 500 cycles at 10 C	233 mAh g^{-1} at 20 C
Adv. Energy Mater. 2013, 3, 428	LVO	280 mAh g^{-1} after 25 cycles at 0.05 C	323 mAh g^{-1} at 0.2 C
Adv. Funct. Mater. 2015. 25, 3497	LVO/C	250 mAh g^{-1} after 2000 cycles at 10 C	106 mAh g^{-1} at 80 C
J. Electrochem. Chem. 2015, 745, 1	LVO/C	398 mAh g^{-1} after 80 cycles at 1C	370 mAh g^{-1} at 15 C
J. Power Sources 2013, 244, 557	LVO	185.55 mAh g^{-1} after 100 cycles at 1 C	249.14 mAh g^{-1} at 1 C
Nano Energy 2015, 12, 709	LVO/G	299 mAh g^{-1} after 1000 cycles at 10 C	88.4 mAh g^{-1} at 50 C
J. Power Sources 2015, 274, 345	LVO/C	394 mAh g^{-1} after 100 cycles at 1 C	100 mAh g^{-1} at 30 C
J. Power Sources 2014, 248, 122	LVO	396 mAh g^{-1} after 100 cycles at 1 C	216 mAh g^{-1} at 4 C
**Our work**	**LVO/C/rGO**	**325 mAh g^{-1}** after **5000 cycles at 10 C**	**230 mAh g^{-1} at 125 C**

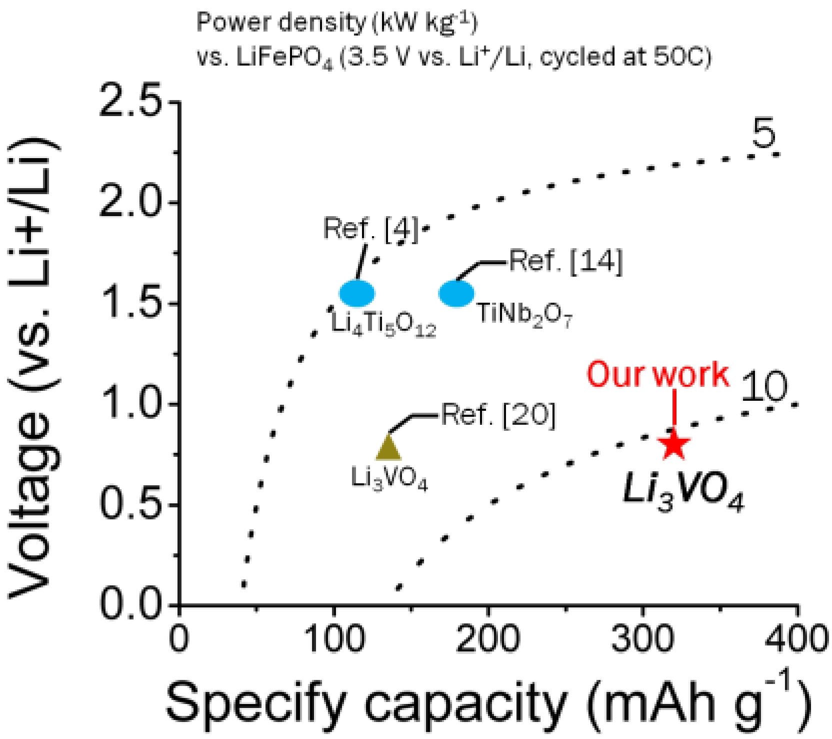

Figure S8. The energy density distribution of the three intercalation/deintercalation anodes, assuming $LiFePO_4$ (100 mAh g^{-1} at 50 C) [ref. S1] as the cathode material.

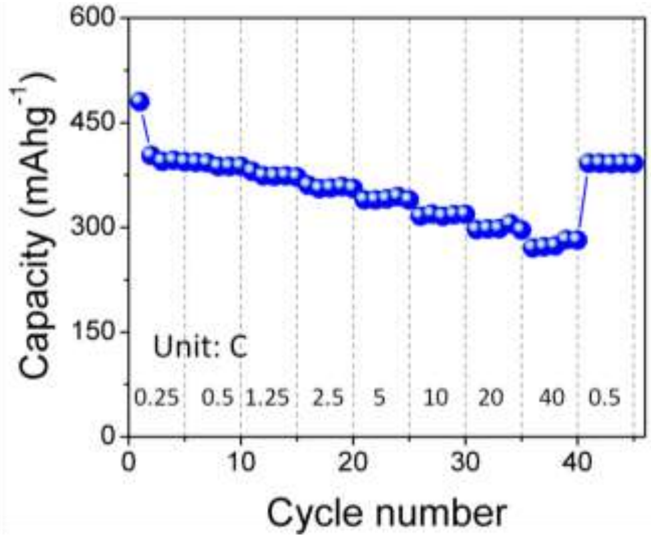

Figure S9. Rate performance of LVO/C/rGO with the composition of active material: acetylene black: binder = 92:3:5 in the composite anode.

References

S1. B. Wang, W. A. Abdulla, D. Wang and X. S. Zhao, *Energy Environ. Sci.*, **2015**,*8*, 869-875
